# Early-Phase Urine Output and Severe-Stage Progression of Oliguric Acute Kidney Injury in Critical Care

**DOI:** 10.3389/fmed.2021.711717

**Published:** 2021-08-11

**Authors:** Haoquan Huang, Xiaohui Bai, Fengtao Ji, Hui Xu, Yanni Fu, Minghui Cao

**Affiliations:** Department of Anesthesiology, Sun Yat-sen Memorial Hospital, Sun Yat-sen University, Guanghzhou, China

**Keywords:** acute kidney injury, intensive care unit, oliguria, urine output, sepsis

## Abstract

**Background:** The relationship between urine output (UO) and severe-stage progression in the early phase of acute kidney injury (AKI) remains unclear. This study aimed to investigate the relationship between early-phase UO^6−12*h*^ [UO within 6 h after diagnosis of stage 1 AKI by Kidney Disease: Improving Global Outcomes (KDIGO) UO criteria] and severe-stage progression of AKI and to identify a reference value of early-phase UO^6−12*h*^ for guiding initial therapy in critical care.

**Methods:** Adult patients with UO < 0.5 ml/kg/h for the first 6 h after intensive care unit (ICU) admission (meeting stage 1 AKI by UO) and UO^6−12*h*^ ≥ 0.5 ml/kg/h were identified from the Medical Information Mart for Intensive Care (MIMIC) III database. The primary outcome was progression to stage 2/3 AKI by UO. After other variables were adjusted through multivariate analysis, generalized additive model (GAM) was used to visualize the relationship between early-phase UO^6−12*h*^ and progression to stage 2/3 AKI by UO. A two-piecewise linear regression model was employed to identify the inflection point of early-phase UO^6−12*h*^ above which progression risk significantly leveled off. Sensitivity and subgroup analyses were performed to assess the robustness of our findings.

**Results:** Of 2,984 individuals, 1,870 (62.7%) with KDIGO stage 1 UO criteria progressed to stage 2/3 AKI. In the multivariate analysis, early-phase UO^6−12*h*^ showed a significant association with progression to stage 2/3 AKI by UO (odds ratio, 0.40; 95% confidence interval, 0.34–0.46; *p* < 0.001). There was a non-linear relationship between early-phase UO^6−12*h*^ and progression of AKI. Early-phase UO^6−12*h*^ of 1.1 ml/kg/h was identified as the inflection point, above which progression risk significantly leveled off (*p* = 0.780). Patients with early-phase UO^6−12*h*^ ≥ 1.1 ml/kg/h had significantly shorter length of ICU stay (3.82 vs. 4.17 days, *p* < 0.001) and hospital stay (9.28 vs. 10.43 days, *p* < 0.001) and lower 30-day mortality (11.05 vs. 18.42%, *p* < 0.001). The robustness of our findings was confirmed by sensitivity and subgroup analyses.

**Conclusions:** Among early-stage AKI patients in critical care, there was a non-linear relationship between early-phase UO^6−12*h*^ and progression of AKI. Early-phase UO^6−12*h*^ of 1.1 ml/kg/h was the inflection point above which progression risk significantly leveled off.

## Introduction

Acute kidney injury (AKI) is common in the intensive care unit (ICU), and mortality dramatically increases as the severer-stage progression of AKI in critical patients (1–3). According to the Kidney Disease: Improving Global Outcomes (KDIGO) guideline, AKI is defined by plasma creatinine criteria or urine output (UO) criteria (4). AKI by KDIGO UO criteria accounts for a large proportion of AKI population (5). In addition, compared with the traditional markers of renal function (e.g., urea nitrogen and creatinine), oliguria is widely regarded as an early marker for early-stage AKI (6, 7).

The therapeutic window of AKI could become narrower as kidney function worsens, and therefore, early improvement in the early phase of AKI is of importance for improving outcome (8–10). However, it has been shown that a number of early-stage AKI patients still suffered from progression to severer stage, even though their UO was above 0.5 ml/kg/h in the early phase of therapy (1). In addition, the latest update of the Surviving Sepsis Campaign guideline does not mention a specific initial therapy goal for UO (11), which was once recommended to be ≥0.5 ml/kg/h in the previous version of the guideline (12). This change indicates that 0.5 ml/kg/h might not be the optimal UO target for therapy in critical patients. Thus, in order to guide the initial therapy for early-stage AKI patients, it is necessary to understand the relationship between UO and progression of AKI in the early phase of AKI. However, there is no study focusing on the relationship between UO and progression of AKI.

The aims of this study were (1) to investigate the relationship between early-phase UO^6−12*h*^ (defined as UO within 6 h after diagnosis of stage 1 AKI by KDIGO UO criteria) and progression of AKI and (2) to identify whether there was an inflection point of early-phase UO^6−12*h*^, above which progression risk significantly leveled off.

## Methods

### Source of Data

The data of this study were extracted from a large US-based critical care database named Medical Information Mart for Intensive Care (MIMIC-III) (13). The MIMIC-III database contains all the ICU data of patients who were admitted to Beth Israel Deaconess Medical Center between 2001 and 2012. The database was approved by the institutional review board (IRB) of Beth Israel Deaconess Medical Center (Boston, MA, USA) and the Massachusetts Institute of Technology (Cambridge, MA, USA). After completing the training course and the Protecting Human Research Participants examination, we have gained access to MIMIC-III (ID: 9786716).

### Participants

**Figure 1:**
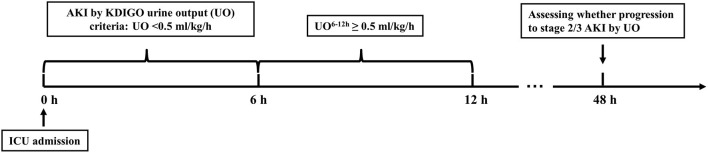
Schematic illustration of the time windows. AKI by KDIGO UO criteria was defined as urine output < 0.5 ml/kg/h for the first 6 h after ICU admission. UO, urine output; UO^6−12*h*^, urine output within 6 h after diagnosis of AKI by KDIGO UO criteria; ICU, intensive care unit; AKI, acute kidney injury; KDIGO, Kidney Disease: Improving Global Outcomes.

All consecutive adult patients (aged ≥ 18 years) who had UO < 0.5 ml/kg/h for the first 6 h (meeting diagnostic criteria of stage 1 AKI by KDIGO UO criteria) (4) and had UO ≥ 0.5 ml/kg/h during the 6-12-h period after ICU admission were screened for possible inclusion in the study (Figure 1). For patients admitted to ICU more than once, only the first ICU admission was included. The exclusion criteria were (1) using any dialysis in the first 24 h after ICU admission and (2) length of ICU stay < 48 h.

### Data Collection

Data including age, gender, weight, ethnicity, comorbidities, sepsis at ICU admission, serum creatinine (Scr) at ICU admission, and the Simplified Acute Physiology Score II (SAPS II) score of the first 24 h were collected from MIMIC-III database. Mean arterial pressure (MAP), vasopressor use, the amount of fluid intake, and UO during the 6-h period after diagnosis of AKI by KDIGO UO criteria were also extracted for analyses. The other fluid losses were initially explored but not included in the final analysis due to their high frequency (>50%) of missing data, included fluid losses in gastrointestinal decompression tube, thoracic drainage tube, and abdominal drainage tube during the 6-h period. Therefore, the fluid output in our study was equal to the amount of UO, and the fluid balance (ml) was defined as the amount of fluid intake (ml) minus the amount of UO (ml) during the 6-h period.

### Outcome

The primary outcome was progression to stage 2 or 3 AKI defined by the KDIGO UO criteria (stage 2: UO < 0.5 ml/kg/h for ≥12 h; stage 3: UO < 0.3 ml/kg/h for ≥24 h or anuria for ≥12 h) at any point in the first 48 h after ICU admission (4).

The secondary outcomes were (1) receiving any dialysis 24 h after ICU admission; (2) length of ICU stay; (3) length of hospital stay; and (4) 30-day mortality since ICU admission.

### Statistical Analyses

While continuous variables were reported as median and interquartile range (IQR), categorical variables were described as whole numbers and percentages. Student's *t*-test or Mann–Whitney *U-*test were used to analyze continuous variables according to their distribution. Fisher's exact test or chi-squared test was used to analyze categorical variables according to their frequencies. Multivariate logistic regression analyses were applied to investigate the effects of UO^6−12*h*^ and the other variables on the occurrence of progression to stage 2/3 AKI by UO. Multicollinearity in regression analyses was detected by variance inflation factor (VIF), with a reference value of 5. Then, multivariate-adjusted model was used to assess the relationship between UO^6−12*h*^ and progression to stage 2/3 AKI by UO. To visualize the relationship between UO^6−12*h*^ and progression to stage 2/3 AKI by UO, generalized additive model (GAM) was used to identify the non-linear relationship. Once the non-linear correlation was observed, a two-piecewise linear regression model was performed to calculate the threshold effect of UO^6−12*h*^ on progression to stage 2/3 AKI by UO in terms of the smoothing plot. When the ratio between progression to stage 2/3 AKI by UO and UO^6−12*h*^ appears obvious in smoothed curve, recursive method automatically calculates the inflection point, where the maximum model likelihood will be used (14, 15). Then the inflection point was selected to dichotomize UO^6−12*h*^.

Sensitivity analyses for the association of UO^6−12*h*^ with AKI stage progression were conducted through the following settings: (1) progression to stage 2 or 3 AKI by KDIGO UO criteria within 7 days after ICU admission as the outcome, (2) progression to stage 3 AKI by KDIGO UO criteria within 48 h or 7 days, and (3) progression to stage 3 AKI by KDIGO Scr criteria within 48 h were employed as the outcomes, separately.

In order to assess the effect size of UO^6−12*h*^ on progression to stage 2/3 AKI by UO in different subgroups, subgroup analyses were carried out in the subgroups as listed: (1) heart failure (yes/no); (2) SAPS II score (<38.95/≥38.95); (3) sepsis at ICU admission (yes/no); and (4) vasopressor use within 6 h after diagnosis of AKI by KDIGO UO criteria (Vasopressor^6−12*h*^) (yes/no). The adjusted odds ratio of UO^6−12*h*^ for progression to stage 2/3 AKI by UO was calculated in each subgroup, and the interaction between UO^6−12*h*^ and subgroups was assessed.

All statistical analyses were performed through R software version 3.4.2 (Institute for Statistics and Mathematics, Vienna, Austria; https://www.r-project.org/). For variables with ≥15% missing values, they were excluded for further analyses. For variables with <15% missing values, they were analyzed using multiple imputation method with “MICE” R package (16). A two-tailed *p*-value < 0.05 was considered to be statistically significant.

## Results

**Figure 2:**
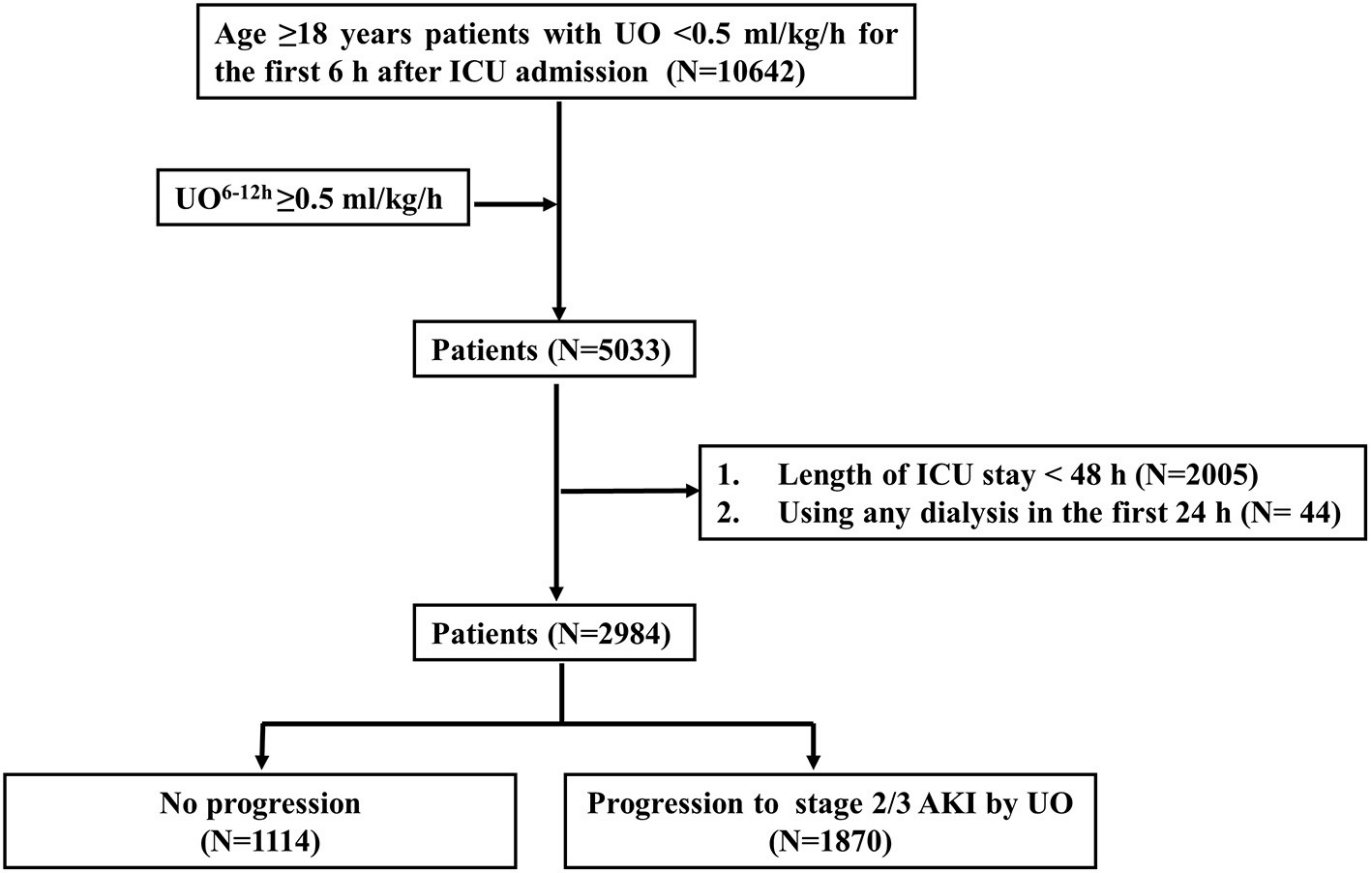
Flowchart of patient selection. UO, urine output; UO^6−12*h*^, urine output within 6 h after diagnosis of AKI by KDIGO UO criteria; ICU, intensive care unit; AKI, acute kidney injury; KDIGO, Kidney Disease: Improving Global Outcomes.

Figure 2 shows the selection process for the study patients. Among the 10,642 adult patients with UO < 0.5 ml/kg/h for the first 6 h after ICU admission, 5,033 patients had UO ≥ 0.5 ml/kg/h during the 6-12-h period after ICU admission. A number of 2,049 patients were excluded because of using any dialysis (*N* = 44) in the first 24 h, or the length of ICU stay <48 h (*N* = 2,005). After selection, 2,984 patients were included for analyses. A total of 1,870 patients had progression to stage 2/3 AKI by UO, and 1,114 patients had no progression.

The differences of patient characteristics are presented in Table 1. Patients with progression to stage 2/3 AKI by UO were more likely to be older (68.37 vs. 65.35 years, *p* < 0.001), have diabetes (32.83 vs. 28.10%, *p* = 0.007), have heart failure (40.53 vs. 28.19%, *p* < 0.001), have hypertension (58.72 vs. 54.58%, *p* = 0.027), have sepsis (54.60 vs. 44.34%, *p* < 0.001), and have higher Scr level at ICU admission (1.00 vs. 0.90 mg/dl, *p* < 0.001) and higher SAPS II score (40.75 vs. 35.93, *p* < 0.001). During the 6-h period after diagnosis of AKI by KDIGO UO criteria, patients with progression to stage 2/3 AKI by UO have higher fluid balance (400.00 vs. 250.00 ml, *p*< 0.001) and lower UO^6−12*h*^ (0.72 vs. 1.08 ml/kg/h, *p* < 0.001).

**Table 1:** Baseline characteristics of participants (*N* = 2,984).

**Characteristic**	**No progression** **(*N* = 1,114)**	**Progression to stage 2/3 AKI by UO** **(*N* = 1,870)**	***P*-value**
Gender male, *n* (%)	663 (59.52)	1,064 (56.90)	0.161
Age, (years; mean ± SD)	65.35 ± 16.21	68.37 ± 14.71	<0.001
Ethnicity, *n* (%)			0.267
Others	295 (26.48)	461 (24.65)	
White	819 (73.52)	1,409 (75.35)	
**Comorbidities**, ***n*****(%)**			
Diabetes	313 (28.10)	614 (32.83)	0.007
Heart failure	314 (28.19)	758 (40.53)	<0.001
Respiratory failure	13 (1.17)	17 (0.91)	0.495
Liver failure	28 (2.51)	67 (3.58)	0.108
Metastatic cancer	60 (5.39)	93 (4.97)	0.621
Hypertension	608 (54.58)	1,098 (58.72)	0.027
Sepsis at ICU admission, *n* (%)	494 (44.34)	1,021 (54.60)	<0.001
Scr at ICU admission [mg/dl; median (IQR)]	(0.70-1.30)	1.00 (0.70-1.50)	<0.001^*^
SAPS II score (mean ± SD)	35.93 ± 12.26	40.75 ± 13.04	<0.001
MAP^0−24*h*^ (mmHg; mean ± SD)	77.58 ± 10.95	76.14 ± 10.10	<0.001
Vasopressor^6−12*h*^, *n* (%)	68 (6.10)	142 (7.59)	0.124
Diuretics^0−24*h*^, *n* (%)	146 (13.11)	256 (13.69)	0.651
**Fluid information during 6–h period after diagnosis of AKI by KDIGO UO criteria**			
Fluid intake [ml; median (IQR)]	700.00 (260.16-1,774.02)	761.67 (235.34-1,875.00)	0.359^*^
Fluid output [ml; median (IQR)]	505.00 (370.00-750.00)	385.00 (291.25-580.00)	<0.001^*^
Fluid balance [ml; median (IQR)]	250.00 (−266.67-1,212.51)	400.00 (−175.00-1,425.50)	<0.001^*^
UO^6−12*h*^ [ml/kg/h; median (IQR)]	1.08 (0.80-1.56)	0.72 (0.59 to 1.03)	<0.001^*^

*UO^6−12h^, urine output within 6 h after diagnosed with AKI by KDIGO urine output criteria; MAP^0−24h^, mean arterial pressure within 24 h after ICU admission; Vasopressor^6−12h^, vasopressor use within 6 h after diagnosis of AKI by KDIGO UO criteria; Scr, serum creatinine; SAPS II score, Simplified Acute Physiology Score (SAPS) II score; KDIGO, Kidney Disease: Improving Global Outcomes*.

* *Mann–Whitney U-test for continuous variables, Fisher's exact for categorical variables with Expects < 10*.

Multivariate logistic regression analysis for progression to stage 2/3 AKI by UO is displayed in Table 2. The fluid output (ml) was excluded from multivariate analysis because its VIF was >5. The remaining variables were selected into the multivariate analysis, including age, gender, ethnicity, hypertension, metastatic cancer, liver failure, respiratory failure, heart failure, diabetes, Vasopressor^6−12*h*^, Diuretics^0−24*h*^, MAP^0−24*h*^, SAPS II score, Scr at ICU admission, sepsis at ICU admission, fluid intake, and fluid balance. It was shown that heart failure, sepsis at ICU admission, SAPS II score, Vasopressor^6−12*h*^, and UO^6−12*h*^ were independently associated with the progression to stage 2/3 AKI by UO.

**Table 2:** Multivariate analysis for progression to stage 2/3 AKI by UO.

**Covariate**	**OR (95% CI)**	***P*-value**
Gender, male	0.96 (0.82, 1.13)	0.654
Age (years)	0.997 (0.991, 1.003)	0.360
Ethnicity, white	1.13 (0.94, 1.36)	0.185
**Comorbidities**		
Diabetes	1.06 (0.89, 1.27)	0.493
Heart failure	1.54 (1.28, 1.84)	<0.001
Respiratory failure	0.61 (0.29, 1.31)	0.264
Liver failure	1.16 (0.72, 1.89)	0.543
Metastatic cancer	0.78 (0.54, 1.12)	0.173
Hypertension	1.10 (0.93, 1.30)	0.264
Sepsis at ICU admission	1.20 (1.02, 1.42)	0.028
Scr at ICU admission (mg/dl)	0.98 (0.89, 1.07)	0.602
SAPS II score	1.023 (1.015, 1.032)	<0.001
MAP^0−24*h*^ (mmHg)	1.00 (0.99, 1.01)	0.690
Vasopressor^6−12*h*^	1.47 (1.06, 2.05)	0.023
Diuretics^0−24*h*^	0.98 (0.78, 1.25)	0.902
**Fluid information during 6-h period after diagnosis of AKI by KDIGO UO criteria**		
Fluid intake (ml)	0.99999 (0.99994, 1.00005)	0.784
Fluid output (ml)	-	-
Fluid balance (ml)	1.00002 (0.99994, 1.00010)	0.680
UO^6−12*h*^ (ml/kg/h)	0.41 (0.36, 0.48)	<0.001

*Variables included in the multivariate analysis: age, gender, ethnicity, hypertension, metastatic cancer, liver failure, respiratory failure, heart failure, diabetes, Vasopressor^6−12h^, Diuretics^0−24h^, MAP^0−24h^, SAPS II score, Scr at ICU admission, sepsis at ICU admission, fluid intake, and fluid balance*.

*UO^6−12h^, urine output within 6 h after diagnosed with AKI by KDIGO urine output criteria; MAP^0−24h^, mean arterial pressure within 24 h after ICU admission; Vasopressor^6−12h^, vasopressor use within 6 h after diagnosis of AKI by KDIGO UO criteria; Scr, serum creatinine; SAPS II score, Simplified Acute Physiology Score (SAPS) II score; KDIGO, Kidney Disease: Improving Global Outcomes*.

**Figure 3:**
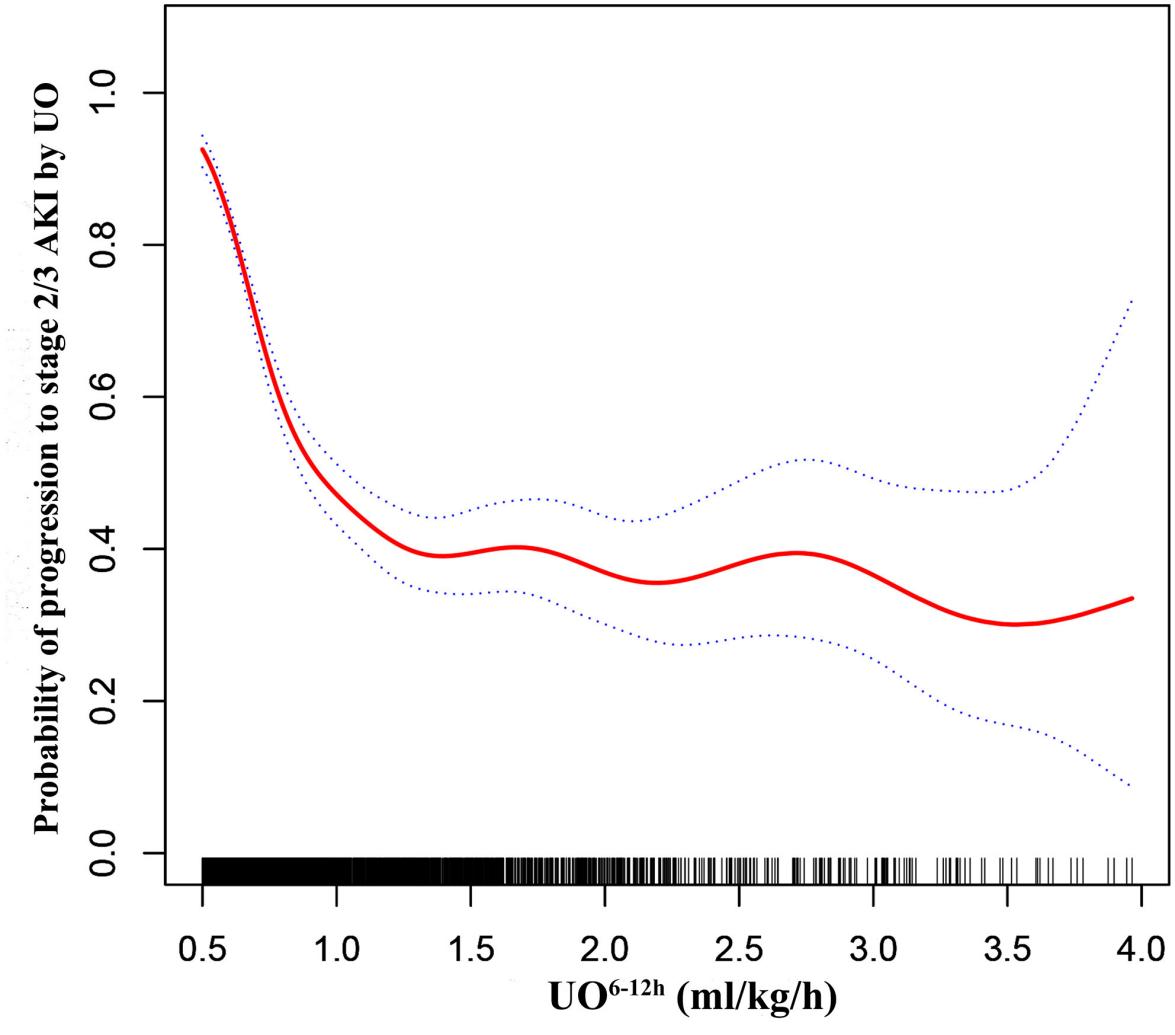
Adjusted association between UO^6−12*h*^ and progression to stage 2/3 AKI by UO. A threshold, non-linear association between UO^6−12*h*^ and progression to stage 2/3 AKI by UO was found in a generalized additive model (GAM). Solid red line represents the smooth curve fit between variables. Blue bands represent the 95% confidence interval from the fit. All adjusted for age, gender, ethnicity, hypertension, metastatic cancer, liver failure, respiratory failure, heart failure, diabetes, Vasopressor^6−12*h*^, Diuretics^0−24*h*^, MAP^0−24*h*^, SAPS II score, Scr at ICU admission, sepsis at ICU admission, fluid intake, and fluid balance. UO^6−12*h*^, urine output within 6 h after diagnosis of AKI by KDIGO UO criteria; MAP^0−24*h*^, mean arterial pressure within 24 h after diagnosis of AKI by KDIGO UO criteria; Vasopressor^6−12*h*^, vasopressor use within 6 h after diagnosis of AKI by KDIGO UO criteria; Scr, serum creatinine; KDIGO, Kidney Disease: Improving Global Outcomes.

GAM was used to visualize the relationship between UO^6−12*h*^ and progression to stage 2/3 AKI by UO (Figure 3). When UO^6−12*h*^ was between 0.5 and 1.0 ml/k/h, the probability of progression to stage 2/3 AKI by UO decreased rapidly as UO^6−12*h*^ increased. When UO^6−12*h*^ was above 1.0 ml/kg/h, the probability almost plateaued with little fluctuation. Subsequently, piecewise linear regression was applied to select the optimal threshold for UO^6−12*h*^. The result showed that 1.1 ml/kg/h was the inflection point for UO^6−12*h*^ after adjusted-multivariate analysis (Table 3), and it was selected to dichotomize UO^6−12*h*^ in the following analyses. Table 3 demonstrates that when UO^6−12*h*^ was ≥0.5–1.1 ml/kg/h, the risk of progression to stage 2/3 AKI by UO reduced significantly by 98% (*p* < 0.001) as UO^6−12*h*^ increased per one unit (ml/kg/h). However, when UO^6−12*h*^ was ≥1.1 ml/kg/h, as UO^6−12*h*^ increased per one unit (ml/kg/h), the risk of progression reduced by 10%, which did not reach statistical significance (*p* = 0.780).

**Table 3:** Threshold effect analysis of UO^6−12*h*^ and progression to stage 2/3 AKI by UO using piecewise linear regression.

**Inflection point of UO^**6–12H**^ (ml/kg/h)**	**OR (95% CI)**	***P*-value**
≥0.5–1.1	0.02 (0.01, 0.03)	<0.001
≥1.1	0.90 (0.77, 1.07)	0.780

*Effect, progression to stage 2/3 AKI by UO; cause, UO^6−12h^*.

*The p-value for logarithmic likelihood ratio test between UO^6−12h^ ≥ 0.5–1.1 (ml/kg/h) and UO^6−12h^ ≥ 1.1 (ml/kg/h) was <0.001*.

*Adjusted for age, gender, ethnicity, hypertension, metastatic cancer, liver failure, respiratory failure, heart failure, diabetes, Vasopressor^6−12h^, Diuretics^0−24h^, MAP^0−24h^, SAPS II score, Scr at ICU admission, sepsis at ICU admission, fluid intake, and fluid balance*.

*UO^6−12h^, urine output within 6 h after diagnosis of AKI by KDIGO urine output criteria; MAP^0−24h^, mean arterial pressure within 24 h after ICU admission; Vasopressor^6−12h^, vasopressor use within 6 h after diagnosis of AKI by KDIGO UO criteria; Scr, serum creatinine; SAPS II score, Simplified Acute Physiology Score (SAPS) II score; KDIGO, Kidney Disease: Improving Global Outcomes*.

In addition, patients with UO^6−12*h*^ ≥1.1 ml/kg/h were prone to have significantly lower rate of receiving any dialysis 24 h after ICU admission (1.14 vs. 2.77%, *p* = 0.008), shorter length of ICU stay (3.82 vs. 4.17 days, *p* < 0.001), shorter length of hospital stay (9.28 vs. 10.43 days, *p* < 0.001), and lower 30-day mortality since ICU admission (11.05 vs. 18.42%, *p* < 0.001).

Sensitivity analyses showed that UO^6−12*h*^ was a significantly independent factor for progression to severer AKI stage by different definitions (Supplementary Table 1). In addition, subgroup analyses showed that the significant impact of UO^6−12*h*^ did not differ among subgroups based on heart failure, sepsis, SAPS II score (<38.95/≥38.95), and sepsis at ICU admission (data not shown).

## Discussion

In this retrospective study, we demonstrated that early-phase UO^6−12*h*^ was a significantly independent variable associated with progression of AKI. As the early-phase UO^6−12*h*^ increased per one unit (ml/kg/h), the risk of progression to stage 2/3 AKI by UO reduced significantly by 60% (*p* < 0.001). More importantly, we found that there was a non-linear relationship between early-phase UO^6−12*h*^ and progression of AKI. Early-phase UO^6−12*h*^ of 1.1 ml/kg/h was the inflection point, above which progression risk significantly leveled off. Those who had early-phase UO^6−12*h*^ ≥ 1.1 ml/kg/h had significant shorter length of ICU stay and hospital stay, as well as lower 30-day mortality since ICU admission. These findings could provide information for guiding initial therapy among early-stage AKI patients in the ICU.

In our study, we focused on early-stage AKI defined by oliguria, because UO is an easy-to-find marker. In addition, previous studies demonstrated that outcomes were worse when patients had AKI by KDIGO UO criteria, even though the Scr level was not elevated (7, 17), indicating that UO is more sensitive than Scr for adverse outcomes. Furthermore, since the “therapeutic window” becomes narrower as kidney injury evolves, a continuous UO monitoring enables clinicians to implement early treatment. Hence, UO is a helpful and critical indicator for early diagnosis and timely treatment for AKI.

Previously, a number of therapies have been reported to attenuate kidney injury among early-stage AKI patients (2, 9, 18–20). However, there is no study focusing on the relationship between UO and progression of AKI in the early phase AKI. In order to carry out more effective treatments, it is necessary for clinicians to understand the relationship between UO and progression of AKI in the early phase of AKI. In this study, the smoothing plot showed that there was a non-linear relationship between early-phase UO^6−12*h*^ and progression of AKI. In addition, early-phase UO^6−12*h*^ of 1.1 ml/kg/h was selected as the inflection point by piecewise linear regression, above which progression risk significantly leveled off. This result indicated that 1.1 ml/kg/h could be a reference value of UO^6−12*h*^ for guiding initial therapies among early-stage AKI patients. In addition, if a patient's UO^6−12*h*^ did not reach 1.1 ml/kg/h, it could remind clinicians to pay more attention to the progression risk and implement more aggressive treatment to avoid progression in the following period.

Sepsis is the leading cause of AKI in the ICU, and septic AKI is associated with poorer outcomes (21). Besides, the most recent Surviving Sepsis Campaign guideline does not mention a UO improvement goal, indicating that the optimal UO recovery value may vary in different sepsis patients. In this study, UO^6−12*h*^ ≥ 1.1 ml/kg/h had significant impacts on progression to stage 2/3 AKI by UO for sepsis group and non-sepsis group (data not shown). Additionally, the adjusted OR for UO^6−12*h*^ was significantly lower (0.25 vs. 0.35, *p* = 0.027) for the sepsis group, indicating that septic AKI patients could have more benefits from the early-phase UO improvement (≥1.1 ml/kg/h) against progression to severer-stage AKI, compared with those without sepsis.

Our study has several strengths. Firstly, the data were extracted from a large critical care database MIMIC-III. Secondly, we employed smoothing plot to directly observe the relationship between UO and AKI progression and to find out the inflection point with clinical implication. Thirdly, our results were robust after subgroup analyses and sensitivity analyses.

There are also some limitations in our study. Firstly, this study is a retrospective study. It is necessary to carry out prospective study to verify the results. Secondly, since the critical patients' conditions are complex, the results could still be confounded by unknown variables, although the confounding factors have been adjusted by multivariate analyses in the study. Thirdly, since there is a lack of information for urinary catheter in MIMIC-III, the urinary catheter type was not included in this study. This could cause bias for the amount of UO collection, because using Foley catheter is more precise than the ordinary catheter. Fourthly, the fluid balance (ml) in our study was defined as the amount of fluid intake (ml) minus the amount of UO (ml) during the 6-h period, since other fluid losses during 6-h period were excluded due to their high frequency of missing data. However, Table 2 shows that neither fluid intake nor fluid balance was independently associated with the progression to stage 2/3 AKI by UO. In addition, we have performed multivariate analysis without adjusting for fluid intake and fluid balance; the main results of our study (i.e., inflection point of UO^6−12*h*^ was 1.1 ml/kg/h) remained robust (data not shown). These results indicated that fluid intake and fluid balance did not affect the main results of our study.

## Conclusion

Among early-stage AKI patients in critical care, early-phase UO^6−12*h*^ was a significantly independent variable associated with progression of AKI, and there was a non-linear relationship between early-phase UO^6−12*h*^ and progression of AKI. Early-phase UO^6−12*h*^ of 1.1 ml/kg/h was the inflection point above which progression risk significantly leveled off; and it could be a reference value for guiding initial therapy among early-stage AKI patients, pending further prospective study.

## Data Availability Statement

Requests to access the datasets should be directed to caomh@mail.sysu.edu.cn.

## Ethics Statement

The study was an analysis of a third-party anonymized publicly available database with pre-existing institutional review board (IRB) approval. Written informed consent for participation was not required for this study in accordance with the national legislation and the institutional requirements.

## Author Contributions

HH designed the study, extracted and analyzed the data, and wrote the first draft of the manuscript. XB reviewed all statistical analyses and critically revised the manuscript. FJ interpreted the data and critically revised the manuscript. HX critically revised the manuscript. YF supervised the analysis of the data and critically revised the manuscript. MC supervised the analysis of the data, critically revised the manuscript, and offered administrative support. All authors have read and approved the final manuscript.

## Conflict of Interest

The authors declare that the research was conducted in the absence of any commercial or financial relationships that could be construed as a potential conflict of interest.

## Publisher's Note

All claims expressed in this article are solely those of the authors and do not necessarily represent those of their affiliated organizations, or those of the publisher, the editors and the reviewers. Any product that may be evaluated in this article, or claim that may be made by its manufacturer, is not guaranteed or endorsed by the publisher.

## Abbreviations

UO^6−12*h*^: urine output within 6 h after diagnosis of stage 1 AKI by KDIGO UO criteria
MAP^0−24*h*^: mean arterial pressure within 24 h after diagnosis of AKI by KDIGO UO criteria
Vasopressor^6−12*h*^: vasopressor use within 6 h after diagnosis of AKI by KDIGO UO criteria
Scr: serum creatinine
AKI: acute kidney injury
ICU: intensive care unit
KDIGO: Kidney Disease: Improving Global Outcomes.
