# Supplementary material for: Early-Phase Urine Output and Severe-Stage Progression of Oliguric Acute Kidney Injury in Critical Care
Source: Front Med (Lausanne). 2021 Aug 11;8:711717. doi: 10.3389/fmed.2021.711717 (PMC8385718; doi:10.3389/fmed.2021.711717)
Supplement: Supplementary file 1 [file Table_1.docx]

Supplementary Material

**Table S1.**Sensitivity analyses for UO^6-12h^≥1.1 ml/kg/h in different models.

| **Model** | **Adjusted OR(95%CI)** | ***P*-value** |
| --- | --- | --- |
| **Progression to stage 2/3 AKI by UO within 48 h after ICU admission as the outcome (Original model)** | 0.28 (0.23, 0.36) | <0.001 |
| **Progression to stage 2/3 AKI by UO within 7 days after ICU admission as the outcome*** | 0.37 (0.24, 0.48) | <0.001 |
| **Progression to stage 3 AKI by UO within 48 h after ICU admission as the outcome** | 0.44 (0.15, 0.87) | 0.035 |
| **Progression to stage 3 AKI by UO within 7 days after ICU admission as the outcome** | 0.31 (0.23, 0.41) | <0.001 |
| **Progression to stage 3 AKI by Scr within 48 h after ICU admissionas the outcome^#^** | 0.58 (0.31,0.72) | <0.001 |

*: If a patient left ICU within 7days after ICU admission, the progression redefined within the ICU stay.

^#^: Minimum of the Scr values available within the 7 days before admission was used as the baseline Scr. When the preadmission Scr was not available, the first Scr measured at admission was used as the baseline Scr.

Adjust for: age, gender, ethnicity, hypertension, metastatic cancer, liver failure, respiratory failure, heart failure, diabetes, Vasopressor^6-12h^, Diuretics^0-24h^, MAP^0-24h^, SAPSII score, Scr at ICU admission, sepsis at ICU admission, fluid intake, fluid balance.

Abbreviations: UO^6-12h^, urine output within 6 h after diagnosis of AKI by KDIGO urine output criteria; MAP^0-24h^, mean arterial pressure within 24h after ICU admission; Vasopressor^6-12h^, Vasopressor use within 6 h after diagnosis of AKI by KDIGO UO criteria; Scr, serum creatinine; SAPS II score, Simplified Acute Physiology Score (SAPS) II score.
